# Inflammatory myopathy in the context of an unusual overlapping laminopathy

**DOI:** 10.20945/2359-3997000000048

**Published:** 2018-05-07

**Authors:** Cristina Guillín-Amarelle, Sofía Sánchez-Iglesias, Antonio Mera, Elena Pintos, Ana Castro-Pais, Leticia Rodríguez-Cañete, Julio Pardo, Felipe F. Casanueva, David Araújo-Vilar

**Affiliations:** 1 Universidad de Santiago de Compostela University of Santiago de Compostela CIMUS IDIS Spain UETeM – Molecular Pathology Group. IDIS-CIMUS, University of Santiago de Compostela, Spain; 2 University Clinical Hospital of Santiago de Compostela Division of Rheumatology Spain Division of Rheumatology, University Clinical Hospital of Santiago de Compostela Spain; 3 University Clinical Hospital of Santiago de Compostela Division of Pathology Spain Division of Pathology, University Clinical Hospital of Santiago de Compostela, Spain.; 4 University Clinical Hospital of Santiago de Compostela Division of Endocrinology and Nutrition Spain Division of Endocrinology and Nutrition, University Clinical Hospital of Santiago de Compostela, Spain; 5 University Clinical Hospital of Santiago de Compostela División of Neurology Spain División of Neurology, University Clinical Hospital of Santiago de Compostela, Spain; 6 CIBER Fisiopatología de la Obesidad y la Nutrición Madrid Spain CIBER Fisiopatología de la Obesidad y la Nutrición (CIBERobn), Madrid, Spain

## Abstract

Laminopathies are genetic disorders associated with alterations in nuclear envelope proteins, known as lamins. The *LMNA* gene encodes lamins A and C, and *LMNA* mutations have been linked to diseases involving fat (type 2 familial partial lipodystrophy [FPLD2]), muscle (type 2 Emery–Dreifuss muscular dystrophy [EDMD2], type 1B limb-girdle muscular dystrophy [LGMD1B], and dilated cardiomyopathy), nerves (type 2B1 Charcot–Marie–Tooth disease), and premature aging syndromes. Moreover, overlapping syndromes have been reported. This study aimed to determine the genetic basis of an overlapping syndrome in a patient with heart disease, myopathy, and features of lipodystrophy, combined with severe metabolic syndrome. We evaluated a 54-year-old woman with rheumatoid arthritis, chronic hypercortisolism (endogenous and exogenous), and a history of cured adrenal Cushing syndrome. The patient presented with a complex disorder, including metabolic syndrome associated with mild partial lipodystrophy (Köbberling-like); mild hypertrophic cardiomyopathy, with Wolff–Parkinson– White syndrome and atrial fibrillation; and limb-girdle inflammatory myopathy. Mutational analysis of the *LMNA* gene showed a heterozygous c.1634G>A (p.R545H) variant in exon 10 of *LMNA*. This variant has previously been independently associated with FPLD2, EDMD2, LGMD1B, and heart disease. We describe a new, *LMNA*-associated, complex overlapping syndrome in which fat, muscle, and cardiac disturbances are related to a p.R545H variant.

## INTRODUCTION

Mutations in the *LMNA* gene (NM_170707.2) have been associated with a broad spectrum of diseases (1), including type 2 familial partial lipodystrophy (FPLD2), LMNA-related metabolic syndrome, type 2 Emery–Dreifuss muscular dystrophy (EDMD2), type 1B limb-girdle muscular dystrophy (LGMD1B), conduction-system diseases and dilated cardiomyopathy (DCM1A), and progeroid syndromes. FPLD2 begins in women during puberty, with a phenotype of fat loss in the limbs and buttocks, fat accumulation in the face and neck, well-defined musculature, phlebomegaly, insulin resistance, atherogenic dyslipidemia, and high cardiovascular risk (2).

The differential diagnosis includes Cushing's syndrome and truncal obesity. Previous studies have described a LMNA-associated metabolic syndrome with a Köbberling-like fat distribution (3,4). EDMD2 is characterized as a progressive skeletal muscle weakness associated with early joint contractures. LGMD1B causes muscular weakness in the spine and pelvic girdle (1).

Cardiac muscle laminopathies can present as nonspecific alterations in electrical conduction, or they can cause malignant arrhythmias and sudden death. Two of the most intriguing features of laminopathies are their clinical heterogeneity and the prevalence of overlapping syndromes. We investigated a female patient with a double Cushing syndrome (endogenous and exogenous), metabolic syndrome, cardiomyopathy, and limb-girdle muscular dystrophy. We aimed to determine whether this syndrome was related to a *LMNA* mutation.

## CASE AND METHODS

This study was approved by the Ethics Review Panel of the Xunta de Galicia. The patient and her relatives provided informed consent for participation in the study and for publication of their clinical, biochemical, and genetic information.

### Patient clinical history

**Figure 1:**
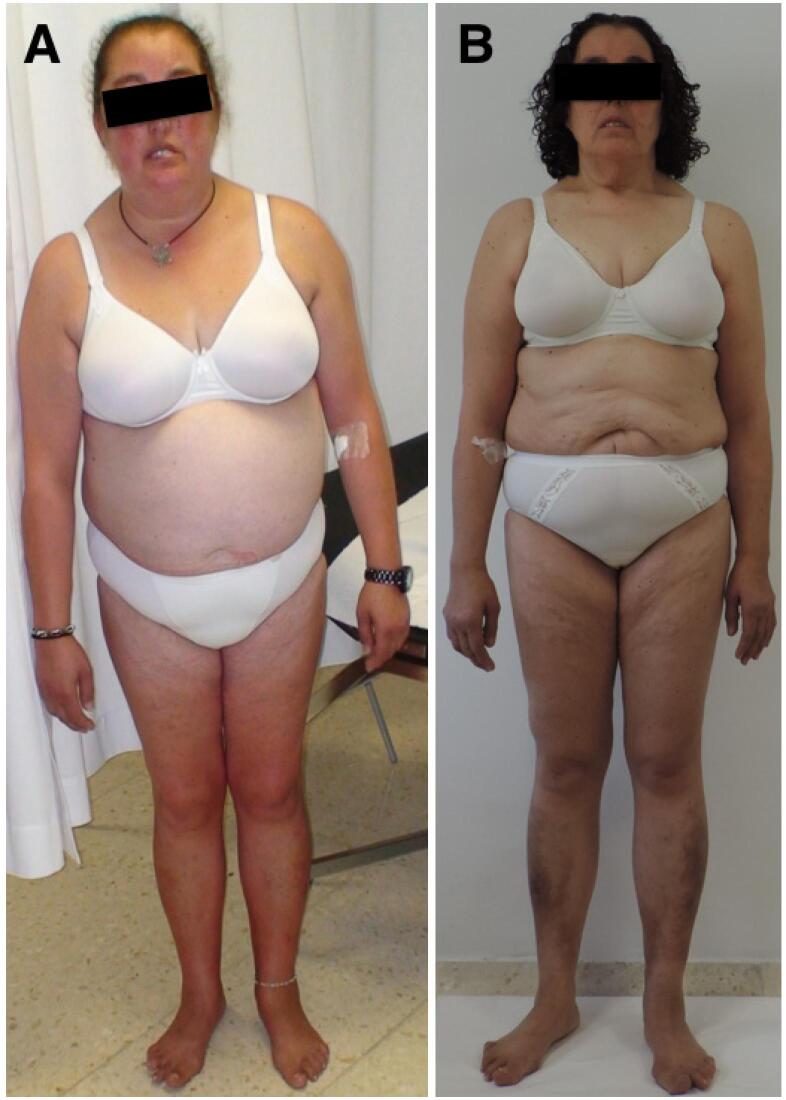
Photographs of the patient show body morphology due to a *LMNA* variant. (**A**) Before receiving a cure for Cushing's syndrome (46 years old). (**B**) Six years after receiving a cure for Cushing's syndrome (54 years old).

The patient (Table 1) was a 45-year-old female diagnosed with rheumatoid arthritis at age 40 y and treated with corticosteroids from that point. She was referred to the Endocrinology Division due to high blood pressure, mixed dyslipidemia, and newly diagnosed diabetes (glycated hemoglobin: 9.6%; systolic blood pressure: 150 mmHg; diastolic blood pressure: 90 mmHg; low-density lipoprotein cholesterol: 5.2 mmol/L; high-density lipoprotein cholesterol: 0.65 mmol/L; triglycerides: 3.42 mmol/L). The patient had had an android appearance since puberty, with mild lipoatrophy in the limbs and buttocks and fat accumulation in the abdomen and face (Figure 1). The patient reported that her deceased father had striking hypermuscular limbs, prominent abdominal fat, and diabetes. He had undergone a pacemaker implantation because of a third-degree atrioventricular block.

**Table 1:** Chronological evolution of clinical data

Age (years)	45	46	47	48	49-52
Event		Adrenal			
	Diabetes. Start	Cushing.		Adrenal	
	antidiabetic	Start statins		surgery,	Myopathy.
	and	and insulin;		start	Stop statins
	antihypertensi	intensive		hydrocortiso	at 49
	ve drugs	antihypertensi		ne	
		ve therapy			
BMI	30.5	31.7	30.8	36.5	25-26
BP (mmHg)	150/90	165/109	130/9	140/93	125/80
			0		
CPK (UI/L)	-	176	88	157	378-2500
LDH (U/L)	-	-	-		556-860
Glucose (mg/dL)	250	331	236	137	65-91
HbA1C (%)	-	9.6	10.2	8.1	6-6.1
Triglycerides (mg/ dL)	164	122	146	254	202-214
HDL (mg/dL)	-	37	41	30	40-40
LDL (mg/dL)	-	161	126	-	63-139
Leptin (µg/L)	-	-	12.7	9.9	1-8.4
Insulin* (mUI/L)	-	46.5	-	-	43.9
UFC (Ug/24h)	-	838	1014	-	36
Cortisol** (ug/dL)	23	30	27.4	2.2	1.6
ACTH	-	13	9	-	60

BP: blood pressure; UFC: urinary free cortisol;

* without exogenous insulin:

** stopping oral corticoid almost 24h before.

At age 46 y, the patient displayed poor glycemic control, and glucocorticoids were discontinued for several months, with no improvement. She was then diagnosed with adrenal Cushing's syndrome, based on a 34-mm right adrenal adenoma (urinary free cortisol: 1100 µg/ 24 h), which was histologically confirmed after a laparoscopic adrenalectomy. Additional hydrocortisone replacement was necessary, and the patient currently continues this treatment. After the adrenal tumor was resected, glycemic control, blood pressure, and lipid levels improved. She continued pharmacological treatment with antihypertensive drugs and metformin, but she was able to discontinue insulin.

Some months after the adrenalectomy, the patient was diagnosed with Wolff–Parkinson–White syndrome associated with atrial fibrillation, due to preexcitation, which was successfully ablated with radiofrequency. Transthoracic ultrasonography revealed a slightly hypertrophic non-dilated left ventricle, with preserved systolic function, type 1 diastolic dysfunction, and mild mitral regurgitation. No coronary lesions were detected with cardiac catheterization.

At age 50 y, the patient complained of muscular weakness without pain or contractures.

Creatine kinase levels ranged from 321 to 2525 IU/L, and high levels persisted after discontinuation of statins. On physical examination, muscle weakness was evident, predominantly in the pelvic girdle. She was unable to get up from the ground and had marked difficulty in rising from a chair without hand support. Electromyoneurography revealed myopathy changes and spontaneous activity (fibrillation and positive waves) in proximal muscles, without polyneuropathy.

### Body composition

Skinfolds (Table 2) were measured in triplicate on the dominant extremity with a Lange skinfold caliper (Cambridge Scientific Industries, MD, USA). Segmental body fat distribution was assessed with whole-body dual-energy X-ray absorptiometry, performed with a Lunar model DPX apparatus (GE Medical Systems, Milwaukee, WI, USA).

**Table 2:** Changes in body composition before and after Cushing cure evaluated by anthropometry and DXA

	Active cushing	Cured cushing	Obese control*	Non obese control*
Age	46	48.6		
Weight (kg)	82.2	65.3		
Height (cm)	161	161		
BMI (kg/m^2^)	31.7	25.21	38.1 ± 6.5	26.6 ± 2.8
Tricipital skinfold (mm)	20	17	38.8 ± 10.0	29.4 ± 11.7
Bicipital skinfold (mm)	15	10	32.4 ± 11.6	21.5 ± 11.8
Suprailiac skinfold (mm)	42	15	52.2 ± 14.7	30.6 ± 14.4
Subescapular skinfold (mm)	35	18	44.2 ± 12.3	28.4 ± 16.4
Thigh skinfold (mm)	19	10	41.1 ± 14.5	22.7 ± 8.6
Calf skinfold (mm)	6	5	18.9 ± 14.2	11.2 ± 4.8
WHR	1.0	0.93	0.89 ± 0.08	0.88 ± 0.06
Total fat (kg)	37.5	20.06	43.8 ± 11.7	26.4 ± 7.7
Total fat (%)	46.1	30.6	48.2 ± 6.4	39.3 ± 7.0
Upper limbs fat (kg)	3.48	2.26	4.44 ± 1.1	3.1 ± 1.0
Upper limbs fat (%)	45.3	31.6	47.7 ± 5.7	42.1 ± 7.3
Lower limbs fat (kg)	9.69	6.16	12.4 ± 3.8	8.4 ± 2.2
Lower limbs fat (%)	40.8	30.5	44.6 ± 6.4	39.8 ± 6.4
Trunk fat (kg)	23.2	10.84	22.9 ± 5.1	14.0 ± 5.7
Trunk fat (%)	51.7	31.8	52.5 ± 7.6	40.8 ± 10.2
Visceral fat (g)	ND	764	2012 ± 894	992 ± 693
Trunkal/Lower limbs fat ratio (kg)	2.39	1.75	1.61	1.66

* Normal ranges from ref. 3; WHR: waist to hip ratio.

### Molecular analyses

DNA was prepared from peripheral white blood cells following standard procedures (5). *LMNA* exons 1–12 and the surrounding intronic sequences were amplified by PCR. Primers and conditions have been previously described (6).

### Histological muscle studies

Deltoid muscle biopsy samples were snap frozen in liquid nitrogen, and cryostat sections were processed and stained to exclude dystrophies and other pathological conditions (e.g., mitochondrial myopathies, metabolic diseases) as follows: PAS, modified Gomori trichrome, Oil Red O, enzyme histochemistry (muscle phosphorylase, muscle phosphofructokinase, lactate dehydrogenase, AMP deaminase, cytochrome-C oxidase, SDH, and NADH), and immunohistochemistry (beta-spectrin to check preservation of the plasma membrane; slow, fast, and fetal myosin; utrophin; alpha, beta, gamma, and delta sarcoglycan; dystrophin fractions: N- terminal, C-terminal, and rod domain; NOS; alpha and beta dystroglycan; dysferlin, caveolin-3, collagen type VI, laminin alpha2 chain of merosin (laminin-2), telethonin, myotilin, desmin, calpain-3, MHC class I antigen, and inflammatory markers (CD68/KP1, CD3 and CD20 for histiocytes and lymphocytes). The only commercial antibodies available for Lamin A/C are not helpful for diagnostic purposes (here it was used as a positive control for emerin antibody).

Immunostains were performed after antigen retrieval using a standard avidin–biotin immunoperoxidase detection technique (EnVision Systems, Dako, Glostrup, Denmark).

## RESULTS

We identified a heterozygous c.1634G>A (p.R545H) variant of *LMNA*, located in exon 10. This variant had not been probed for pathogenicity with in silico approaches (PolyPhen and SIFT).

**Figure 2:**
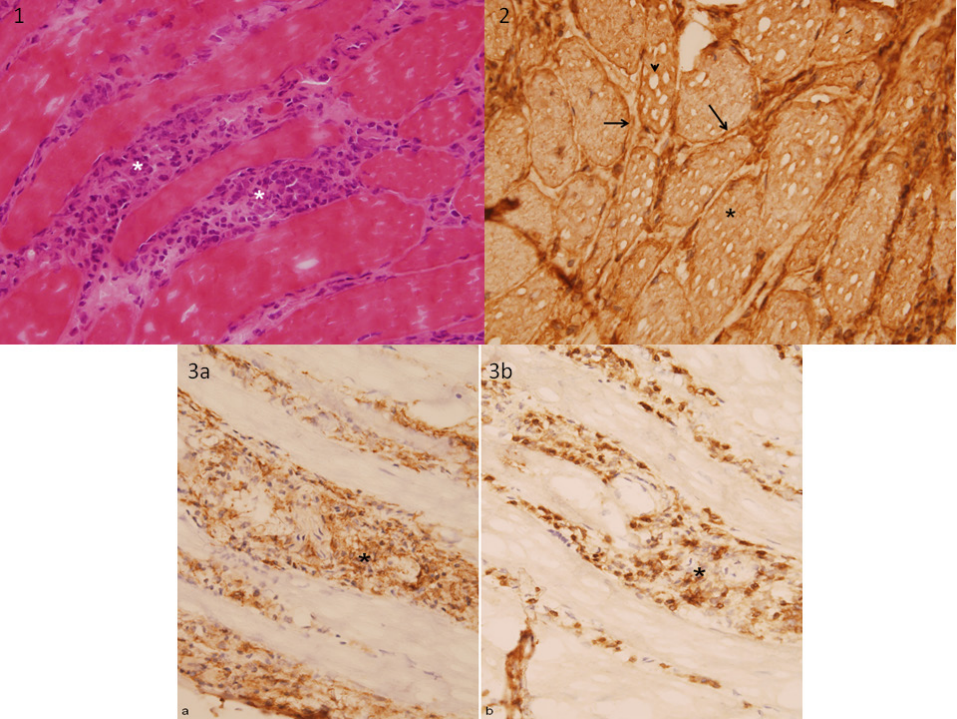
(**1**) Snap frozen cryostat sections of deltoid muscle showing endomisial inflammatory infiltrates composed mainly of hystiocytes (asterisks) (Hematoxylin and eosin, 400x). (**2**) MHC class I antigen is upregulated in all fibers, with immunolabeling at plasma membrane (arrow) and sarcoplasm (asterisk). See also “pseudovacuoles” secondary to “ice crystals” snap frozen artifact (arrow head) (Immunoperoxidase reaction, diaminobenzidine brown staining chromogen, 400x). (**3a**) Immunostaining of CD68 (KP1) antibody remark histiocityc inflammatory component between myofibers, with (**3b**) a minor population of CD3 antibody positive T lymphocytes (asterisks) (400x).

Its allelic frequency was 0.00019 (1000 Genomes Project). Of the patient's relatives whom we studied (mother, sisters, offspring), none carried this variant. Histological findings of muscle samples were consistent with acute and chronic, nonspecific, inflammatory myopathy (Figure 2).

## DISCUSSION

We investigated an unusual case of laminopathy and found that it was due to a p.R545H *LMNA* variant that overlapped with chronic hypercortisolism. Thus, after chronic hypercortisolism had been diagnosed and cured, the patient exhibited atypical partial lipodystrophy, idiopathic inflammatory myopathy, and cardiomyopathy with conduction disturbances. Although this variant has not been shown to be pathogenic with in silico approaches, it has been associated with FPLD2 (7), EDMD2 (8), LGMD1B (9), and heart disease (10).

In FPLD2, more than 80% of cases are due to missense mutations in exon 8; however, atypical phenotypes have been related to mutations in other exons (11). Moreover, several patients with mutations outside the immunoglobulin-like fold of lamin A have lacked the typical FPLD2 phenotype but experienced insulin resistance (4). In addition, some cases of FPLD2 have been associated with heart conduction disorders, valvulopathies, and cardiomyopathy (12-14). Other authors have reported LMNA-associated complex phenotypes, including heart failure and limb-girdle muscular dystrophy, due to a Ser334del variant (15,16); muscular dystrophy, lipodystrophy, and cardiac rhythm disturbances related to a R527P variant (17); or FPLD, early heart failure, first-degree atrioventricular block, and late proximal muscle weakness due to a R28W variant (12).

Exon 10 of *LMNA* corresponds to the C-terminal domain, common to both lamins A and C, which forms an immunoglobulin-like, three-dimensional structure (1). The conformation of this domain is well defined for residues 430–545 (18). Arginine 545 is at the external surface of the structure. Mutations in the Ig-fold can affect either head-to-tail polymerization, which destabilizes the three-dimensional structure of the C-terminal domain, or lamin A/C interactions with other proteins (1). At least 13 missense mutations at the C-terminal domain are related to EDMD2 (18), including the nearby R541H. Although the R545 residue has not been specifically studied, it does not seem to influence the stability of the 3D structure. However, the exchange of arginine (positive polar) for histidine (neutral polar) could alter interactions with other proteins at the nuclear lamina.

This study was particularly challenging because of the presence of Cushing's syndrome and the autoimmune background. Chronic hypercortisolism causes a characteristic fat distribution, in particular, excess abdominal fat (19); however, no particular changes in limb fat have been reported (20). Strikingly, in this patient, once hypercortisolism was cured, an abnormal fat distribution became more evident, although it was not as severe as observed in classical Dunnigan disease. This change in fat distribution was particularly intriguing because it highlighted important differences among *LMNA* mutations that cause FPLD (13). The R545H variant, previously associated with FPLD (7), caused severe metabolic syndrome with android fat distribution in this patient.

The patient was initially diagnosed with polymyositis, based on girdle weakness, high creatine kinase, and muscle lymphocyte infiltration. Because of her autoimmune background, this diagnosis was probably the most parsimonious. However, the R545H variant has been associated with LGMD1B (9), which clinically overlaps with polymyositis. Other *LMNA* mutations that cause muscular and/or cardiac laminopathies have been related to inflammatory changes in muscle specimen biopsies. For example, in biopsies from patients with infantile- onset LMNA-associated myopathy, Komaki and cols. (21) reported mononuclear cell infiltrations that were positive for lymphocyte markers CD4, CD8, or CD20, active necrosis, and regeneration. Additionally, they observed elevated sarcolemmal HLA staining in many fibers. Similarly, HLA class I antigens were upregulated in our samples and in samples described previously in a study on LGMD1B (15,16). It cannot be ruled out that chronic hypercortisolism had influenced the myopathy. However, there are some clues to differences between the corticoid-myopathy case and the case described here.

In the first case, serum values of muscle enzymes are typically normal or slightly elevated, electromyoneurography is usually normal, and there are no inflammatory infiltrates or necrosis on muscle biopsy samples (22). Although unusual, polymyositis has been associated with cardiac involvement, manifested as rhythm disturbances, conduction defects, and heart failure (23). Similarly, laminopathies, like FPLD2 or LGMD1B (9,14), and particularly the R545H variant, have also been related to heart disease (10). Patients with cardiac compromise related to *LMNA* typically show initial signs of nonspecific rhythm disturbances after age 30 y (24), and they frequently need a permanent pacemaker. Later, cardiac compromise may be further complicated with cardiomyopathy (25). Our patient was diagnosed with Wolff–Parkinson–White syndrome, atrial fibrillation, mitral regurgitation, and mild hypertrophic cardiomyopathy. Taken together, the diagnoses of severe metabolic syndrome, girdle weakness appearing in the fourth decade, and heart involvement in a patient that carried the R545H variant in *LMNA* resembled a complex laminopathic disorder. However, the presence of chronic hypercortisolism represented a confounding factor, and rheumatoid arthritis prevented us from definitely ruling out a random association of different unrelated autoimmune entities.

In summary, this case emphasizes the need for actively searching for *LMNA* mutations in patients with clinical features compatible with partial lipodystrophy, cardiac conduction abnormalities, cardiomyopathy, and/or certain types of myopathy. A correct diagnosis can facilitate early interventions to prevent the consequences of these pathologies, which can be lethal.
